# Protocol for Conducting Scoping Review on Reliability and Validity of Low-Stakes Assessments in Medical Education

**DOI:** 10.12688/f1000research.158787.1

**Published:** 2024-11-22

**Authors:** Imran Zafar, Susan Waller, Lambert Schuwirth, Carvalho Filho, Mohi Eldin Magzoub

**Affiliations:** 1 Medical Education, United Arab Emirates University, Al Ain, Abu Dhabi, United Arab Emirates; 2 Medical Education, Flinders University, Adelaide, South Australia, Australia; 3 Medical Education, University of Groningen, Groningen, Groningen, The Netherlands

**Keywords:** Low-stakes assessment, validity, reliability

## Abstract

Low-stakes assessments (LSAs) are widely used in medical education to promote continuous learning by providing formative feedback and minimizing the high-stress environment associated with high-stakes assessments. While LSAs are recognized for their role in supporting student learning, questions remain about their reliability and validity. This scoping review aims to systematically identify and synthesize existing literature on the reliability and validity of LSAs within medical education contexts. This study aims to map the methodologies used to establish psychometric parameters, identify problems, and offer best practices.

The review will use the Joanna Briggs Institute methodology, encompassing a comprehensive search across six academic databases and grey literature to identify relevant studies published from 2000 onward. Two reviewers will independently screen and extract data, using the Covidence tool for systematic screening and data extraction. Data will be analyzed using qualitative and quantitative approaches to provide an overview of reliability and validity evidence for LSAs in medical education. Insights into motivational and psychometric theories, such as Self-Determination Theory, Classical Test Theory, and Generalizability Theory, that inform LSA design and implementation will be used to analyse the findings in this review.

Expected outcomes include a detailed map of the literature on LSA reliability and validity, identification of key challenges, theoretical underpinnings and recommended strategies for improving LSA practices in medical education. Findings will inform future research and provide guidelines for optimizing the use of LSAs to enhance student learning outcomes, assessment integrity, and the educational experience in medical training.

## Rationale and background

Low-Stakes Assessments (LSAs) in medical education are essential tools that support continuous learning by providing formative feedback and monitoring student progress. These assessments are designed to minimize the anxiety and pressure often associated with high-stakes evaluations, thereby creating an environment conducive to authentic learning and self-regulation. The reliability of LSAs is grounded in their ability to consistently provide accurate feedback that reflects students’ actual learning and competencies, while their validity is ensured by aligning the assessments closely with educational objectives and learning outcomes ( Schut et al., 2018).

One of the key strengths of LSAs is their capacity to foster a continuous flow of information, allowing learners to self-regulate their learning effectively. Schut et al. (2018), emphasized that the formative nature of these assessments enhances their validity by ensuring that the feedback provided is relevant and actionable. Reduced stakes associated with these assessments contribute to their reliability, as they encourage more authentic student engagement and reduce test anxiety, leading to more accurate assessments of student knowledge and skills ( Ganesan et al., 2023).

The design of LSAs also supports the provision of honest and constructive feedback, which is crucial for maintaining the validity of the assessment process. It is argued that using multiple LSAs throughout the educational process allows for a more comprehensive evaluation of student performance, thereby increasing the reliability of the overall assessment system ( Schut et al., 2020). This approach mitigates the risk of a single assessment unduly influencing final outcomes, ensuring that students are evaluated based on a broader and more representative sample of their abilities.

Despite the benefits, challenges remain in ensuring the reliability and validity of LSAs. Factors such as student motivation and the novelty of assessment formats can impact performance, affecting the reliability of these assessments ( Madrazo et al., 2018). Research by Silm et al. (2013) and Knekta & Eklöf (2015) highlights the importance of understanding test-taker motivation to maintain both the reliability and validity of LSAs, particularly in medical education, where consistent and accurate assessment of competencies is critical.

## What are the types of low-stakes assessment in medical education?

LSAs in medical education encompass a variety of formats and methodologies designed to facilitate learning and provide feedback without the high-pressure consequences associated with traditional high-stakes assessments. These assessments are integral to fostering a supportive educational environment and can take several forms, each serving distinct purposes in the learning process.

1. Formative Assessments: These assessments are designed to monitor student learning and provide ongoing feedback that can be used by instructors to improve their teaching and by students to enhance their learning. Formative assessments, particularly when they are low-stakes, create opportunities for self-regulation and continuous improvement among learners ( Schut et al., 2020). Examples of LSAs in this category include quizzes, practice exams, and informal assessments during clinical rotations.
2. Objective Structured Clinical Examinations (OSCEs): While OSCEs can be high-stakes, they are often utilized in a low-stakes format to assess clinical skills in a supportive environment. Low-stakes OSCEs can help students gauge their competencies and identify areas for improvement without the pressure of high-stakes evaluations ( Madrazo et al., 2018). This format allows for practical skill assessment in a controlled setting, promoting learning through practice.
3. Peer Assessments: Peer evaluations can give and receive feedback between peers. This method encourages collaborative learning and reflection. Peer assessments can motivate students and enhance their learning experience in low-stakes contexts ( Schüttpelz-Brauns et al., 2020).
4. Self-Assessments: Self-assessment tools enable students to evaluate their own knowledge and skills, fostering a sense of ownership over their learning. These assessments can help identify gaps in knowledge and encourage students to take proactive steps toward improvement. Self-regulated learning is significantly influenced by the stakes associated with assessments ( Ganesan et al., 2023).
5. Progress Committees: The use of progress committees introduces an independent third party into the assessment process, allowing for a more holistic view of a student’s progress over time. This approach can help mitigate the pressure associated with individual assessments and provide a supportive framework for student development ( Schut et al., 2020).
6. Reflective Journals: Encouraging students to maintain reflective journals can promote critical thinking and self-reflection. This method allows students to document their learning experiences, challenges, and growth throughout their medical education journey ( Paloniemi et al., 2024).
7. Quizzes and Short Tests: Frequent low-stakes quizzes can help reinforce learning and provide immediate feedback. These assessments can be administered in various formats, such as online quizzes or in-class tests, and are designed to assess knowledge retention and understanding of key concepts ( Bains et al., 2023).

## Importance of reliability and validity

The reliability and validity of LSAs are critical in ensuring that these assessments accurately reflect student abilities and knowledge. Reliable assessments consistently yield the same results under similar conditions, while valid assessments accurately measure what they are intended to measure. Without these qualities, LSAs cannot effectively inform educational decisions or contribute to meaningful learning.

The importance of reliability and validity in LSAs within medical education cannot be overstated.

Reliability in low-stakes assessments is essential for maintaining the integrity of the evaluation process. According to Schut et al., reliable assessments provide consistent results across different contexts and times, which is particularly important in medical education where competencies must be accurately gauged to ensure patient safety and effective care ( Schut et al., 2020). These assessments, while designed to be less stressful for students, still require rigorous standards to ensure that they effectively measure the competencies and knowledge of learners. Both reliability and validity are crucial for ensuring that LSAs contribute positively to the educational process.

Reliable LSAs can help educators identify areas where students may need additional support, thereby enhancing the overall learning experience ( Schüttpelz-Brauns et al., 2020). The reliability of assessment tools can be bolstered through careful design and the use of multiple assessors, in a programmatic approach that incorporates various low-stakes assessments to provide a more comprehensive evaluation of student performance ( Schut et al., 2020).

Validity, on the other hand, ensures that the assessments are measuring the intended constructs. The necessity of establishing validity evidence for assessment tools in medical education is integral to ensuring that the results of these assessments accurately reflect student competencies ( Hoover et al., 2013). This is particularly relevant in LSAs, where the goal is to foster learning rather than merely to evaluate.

The interplay between reliability and validity is critical. If an assessment is not reliable, it cannot be valid and inconsistent results undermine the credibility of the assessment process.

## Theoretical framework for assessing the reliability and validity

The theoretical framework for assessing the reliability and validity of LSAs in medical education is multifaceted and encompasses several psychometric theories and motivational frameworks. It emphasizes the importance of structured assessment frameworks that align with educational outcomes, thereby enhancing the reliability and validity of the assessments used ( Pearce et al., 2015). The concept of validity in medical education has evolved, necessitating a consensus on its definition and application within assessments ( Royal, 2017). This is particularly relevant for LSAs, which often face challenges related to students’ test-taking behaviors and motivation ( Royal, 2017). Strategies that promote serious engagement, such as mentorship discussions and clear consequences for non-participation, can enhance the effectiveness of these assessments ( Royal, 2017). Thus, integrating these elements into a cohesive framework can significantly improve the quality and impact of low-stakes assessments in medical education ( Schuwirth et al., 2022).

## Key theories in assessment

### Motivational theories

Understanding student motivation is crucial for enhancing the effectiveness of LSAs. The following theories provide insights into how to increase student engagement and effort during these assessments:

1. Self-Determination Theory (SDT) emphasizes the role of intrinsic motivation and the need for autonomy, competence, and relatedness. Strategies derived from SDT, such as providing feedback and discussing performance with mentors, have been shown to increase the seriousness with which students approach LSAs ( Deci & Ryan, 1985; El Boghdady & Alijani, 2017).
2. Expectancy-Value Theory posits that students’ motivation is influenced by their expectations of success and the value they place on the task. Incorporating elements that enhance the perceived value of LSAs can lead to increased engagement and effort from students ( Schüttpelz-Brauns et al., 2018).

### Psychometric theories and theoretical frameworks

1. Classical Test Theory (CTT) focuses on the reliability of test scores, emphasizing the consistency of measurements across different instances. In CTT, observed scores are composed of true scores and measurement error, making it crucial for ensuring that LSAs yield reliable results( Schuwirth & van der Vleuten, 2011).
2. Generalizability Theory (GT) extends CTT by examining how various sources of error affect test scores. GT allows for a more nuanced understanding of reliability by considering multiple facets of measurement, such as different raters or test forms, which is particularly relevant in LSAs where variability in student engagement can influence results ( Schuwirth & van der Vleuten, 2011). G-Theory can be applied to performance-based assessments, allowing educators to evaluate the reliability of their assessments more rigorously ( Peeters, 2021). This approach can help identify areas for improvement in assessment design and implementation.
3. Item Response Theory (IRT) analyzes the relationship between an individual’s latent traits (abilities) and their item responses. It is beneficial for developing assessments that can adapt to different levels of student ability, thereby enhancing the validity of low-stakes assessments by ensuring they accurately reflect student knowledge ( Schuwirth & van der Vleuten, 2011).
4. Validity Theory: Validity encompasses the degree to which an assessment measures what it is intended to measure. The framework for establishing validity can be based on the five sources of validity evidence proposed by the Standards for Educational and Psychological Testing. These sources include content validity, criterion-related validity, construct validity, consequential validity, and face validity, providing preliminary validity evidence for LSAs, particularly in clinical settings, ensuring assessments accurately reflect the competencies being measured ( Sireci & Rodriguez, 2022). This evidence is critical for building trust in the assessment process among students and educators alike.
5. Programmatic Assessment: The concept of programmatic assessment integrates multiple assessment methods to provide a comprehensive evaluation of student competencies. Multiple LSAs, especially those without numerical grades, can enhance the reliability and validity of the assessment process ( Schut et al., 2020). This approach allows for a more holistic view of student performance and encourages continuous feedback, which is essential for fostering a learning-oriented environment.
6. Feedback and Learning Theory: The role of feedback in the assessment process is crucial for enhancing learning outcomes. There is a catalytic effect of assessment, where feedback generated from assessments leads to improved learning and competency development ( Heeneman et al., 2015) This aligns with the principles of formative assessment, which emphasize the importance of using assessment as a tool *for* learning rather than merely *of* learning.
7. Psychometric principles are vital for ensuring the reliability and validity of LSAs. These include the careful design of assessment items, the training of raters, and the use of statistical methods to analyze assessment data. In simulation-based assessments rater training has been shown to enhance both reliability and validity ( Mackenzie et al., 2023). Ensuring that raters are well-trained can mitigate biases and improve the consistency of assessment outcomes.
8. Student Perceptions and Motivation: The perceptions of students regarding the stakes of assessments can significantly influence their engagement and performance ( Schut et al., 2018). Understanding the psychological dynamics at play can help educators design assessments that motivate students and encourage genuine learning.

### Application to LSAs

Elements of the above theories will be applied to examine how various factors, such as item design, scoring methods, and test administration conditions, influence the reliability and validity of LSAs. The review will particularly consider how the principles of CTT and IRT can be adapted to the unique context of LSAs in medical education.

## Methods

The review will use the Joanna Briggs Institute’s framework for scoping review studies, searching six databases and grey literature. A presearch will be done in PubMed, Scopus, and Google Scholar using terms related to LSAs, formative assessment, continuous assessment, and programmatic assessment within the context of medical education. The Covidence Systematic Review tool will aid in screening and conflict resolution.

The reference lists of included studies will be checked manually for other relevant literature. Two research team members will independently screen and extract data, resolving discrepancies with a third team member. Inclusion and exclusion criteria will be refined iteratively based on key research themes.

Data will be qualitatively and quantitatively analyzed and presented in diagrams or tables with a narrative synthesis. The synthesis will map data related to low-stakes assessment in medical education, identify challenges and opportunities of this assessment approach. The review will follow PRISMA guidelines, focusing on the reliability and validity of the LSAs in medical education.

### Research questions

— What are the most used methods for establishing the reliability and validity of LSAs in medical education?
— How do different factors, such as test format, item type, and scoring procedures, affect the reliability and validity of LSAs?
— What are the challenges in maintaining reliability and validity in low-stakes assessments, and how are these challenges addressed in the literature?
— What best practices are recommended for enhancing the reliability and validity of LSAs in medical education?
— What are the underpinning theories, frameworks or models cited in the literature which support the reliability and validity of LSAs?

### Inclusion/Exclusion criteria

**Inclusion criteria**:

1. Topic: The study must primarily focus on LSAs’ reliability and validity in medical education.
2. Study Population: Participants should include medical students, interns, or residents (junior doctors in training) completing LSAs.
3. Assessment Type: Assessments must be low-stakes, meaning they do not significantly affect final grades or academic standing.
4. Publication Date: Only studies published after January 2000 will be considered.
5. Language: Studies must be published in English.
6. Methodology: Studies employing quantitative, qualitative, or mixed-methods approaches are eligible.

**Exclusion criteria**:

1. High-Stakes Assessments: Studies focused on high-stakes assessments that significantly impact grades or academic standing will be excluded.
2. Non-Medical Education: Studies from non-medical education settings or involving non-medical students will be excluded.
3. Non-English Language: Studies not published in English will be excluded.
4. Non-Primary Focus on Reliability and Validity: Studies that do not explicitly investigate LSAs’ reliability and validity will be excluded.
5. Pre-2000 Publications: Studies published or grey literature dated before 2000 will not be included.

### Study types

The review will consider a wide range of study types, including quantitative research (e.g., randomized controlled trials, quasi-experimental studies, cohort studies, cross-sectional studies, and pre-post studies), qualitative research (e.g., interviews, focus groups, and case studies), and mixed-methods studies that combine both data types. Systematic reviews, meta-analyses, and surveys will also be included to capture broader trends, summarize existing research, and highlight gaps.

### Scoping review proposal

This scoping review will adhere to the Joanna Briggs Institute (JBI) methodology, specifically utilizing the PCC framework (Population, Concept, and Context) to clearly define the review’s focus. In line with the JBI Scoping Review checklist, the review will incorporate these elements explicitly to ensure comprehensive coverage and alignment with the protocol requirements. Additionally, the review will follow the guidelines of the Preferred Reporting Items for Systematic Reviews and Meta-Analyses extension for Scoping Reviews (PRISMA-ScR). The planned start date for this review is December 2024, with an expected completion date in March 2025.

### Database selection

The review will search six key databases, including PubMed, MEDLINE, ERIC, PsycINFO, Scopus, and Web of Science.

### Search strategy

A detailed search strategy will be developed with the guidance of a medical librarian, using a combination of keywords and Medical Subject Headings (MeSH) terms relevant to LSAs, student learning, and medical education. Boolean operators (AND, OR, NOT) will be used to refine the search results.

### Screening process

An initial screening of titles and abstracts will be conducted by two independent reviewers to identify studies that meet the inclusion criteria. Studies passing this screening will undergo a full-text review to confirm their relevance.

### Data extraction

Data extraction will be conducted using a standardized form to systematically capture essential information from each included study. This form will record details such as study title, authors, publication year, objectives, study design, theoretical framework, participant characteristics, types of low-stakes assessments (LSAs) used, outcome measures related to student learning, and key findings. To ensure accuracy, two reviewers will independently extract data, resolving any discrepancies through discussion or, if needed, consultation with a third reviewer. Extracted data will be presented in a summary table and accompanied by a narrative explanation.

### Expected outcomes

— A comprehensive map of the current literature on the reliability and validity of LSAs in medical education.
— Identification of common challenges and effective strategies for enhancing reliability and validity in LSAs.
— Recommendations for future research directions related to the validity and reliability of LSAs, based on identified gaps.
— Underpinning theories associated with reliability and validity of LSAs.

## Ethics and dissemination

As this review will involve only published, and grey literature, no ethical approval is required. Findings will be disseminated through academic conferences and publications in peer-reviewed journals.

## Data Availability

No data are associated with this article. Repository: PRISMA-P checklist for ‘Protocol for conducting scoping review on reliability and validity of low-stakes assessments in medical education’. DOI: 10.6084/m9.figshare.27627834.v2 ( Zafar et al., 2024). Data are available under the terms of the Creative Commons Zero “No rights reserved” data waiver (CC0 1.0 Public domain dedication).
