# Supplementary material for: Protocol for Conducting Scoping Review on Reliability and Validity of Low-Stakes Assessments in Medical Education
Source: F1000Res. 2024 Nov 22;13:1413. [Version 1] doi: 10.12688/f1000research.158787.1 (PMC12171711; doi:10.12688/f1000research.158787.1)
Supplement: Peer review history

# Reviewer report: Reviewer response for version 1

**DOI:** 10.5256/f1000research.174426.r386376

**Published:** 2025-06-13

**Authors:** Julie A Hunt

**Affiliations:** 1 Lincoln Memorial University, Harrogate, Tennessee, USA

The authors are commended for undertaking a review of reliability and validity of low-stakes assessments (LSAs) in medical education. This is an important topic affecting medical education, impacting students' grades as well as the feedback that influences their growth as future professionals.

 The authors have done a good job in providing a rationale for the need for the study and in describing the importance of reliability and validity.

 The authors could improve upon the list of LSAs performed in medical education. Some of those listed are not LSAs (progress committees), and some common LSAs are missing (e.g. in-laboratory assessments of clinical skills).

 In the review of theoretical frameworks for assessing reliability and validity, I would have expected to have seen Michael Kane's work on argument-based validation cited.

 In key theories in assessment, the authors stated the 5 sources of evidence proposed by the Standards for Educational and Psychological Testing (under "4. Validity Theory") incorrectly. They are content evidence, response process evidence, internal structure evidence, relationship with other variables evidence, and consequences evidence. These have replaced the outdated sources cited in the text.

 The authors are commended for a robust review of the assessment, reliability, and validity literature underpinning their study, and for including qualitative studies in their methods, as these are often overlooked. The methods did a good job of describing the search strategy but should have included a list of the intended search terms as an appendix.

Is the study design appropriate for the research question?

Yes

Is the rationale for, and objectives of, the study clearly described?

Yes

Are sufficient details of the methods provided to allow replication by others?

No

Are the datasets clearly presented in a useable and accessible format?

Not applicable

Reviewer Expertise:

Educational research including clinical skills model and rubric validation and student skills assessment

I confirm that I have read this submission and believe that I have an appropriate level of expertise to confirm that it is of an acceptable scientific standard.

# Reviewer report: Reviewer response for version 1

**DOI:** 10.5256/f1000research.174426.r355767

**Published:** 2025-01-20

**Authors:** Eva Mukurunge

**Affiliations:** 1 University of the Free State, Bloemfontein, Free State, South Africa

A well written protocol for an essential study in assessment in Medical education.

 You need to revise your list of types of Low-Stakes Assessments (LSAs) in the view that assessments are generally either formative or summative. You have listed formative assessments as an example of LSAs which then limits your list since all LSAs fall under formative assessments.

 In order to clear the confusion, a definition of what you are referring to as LSAs would be essential.

 Your list of LSAs includes Progress Committees, which should rather be mentioned as quality assurance checks in the implementation of LSAs.

Is the study design appropriate for the research question?

Yes

Is the rationale for, and objectives of, the study clearly described?

Yes

Are sufficient details of the methods provided to allow replication by others?

Yes

Are the datasets clearly presented in a useable and accessible format?

Not applicable

Reviewer Expertise:

Assessment in Medical education; Communication in Medical Education

I confirm that I have read this submission and believe that I have an appropriate level of expertise to confirm that it is of an acceptable scientific standard.

## References

[1] : Understanding the fundamental differences between formative and summative assessment. *Global Scientific and Academic Research Journal of Education and literature* . Reference source
